# Supplementary material for: Insight into One Health Approach: Endoparasite Infections in Captive Wildlife in Bangladesh
Source: Pathogens. 2021 Feb 23;10(2):250. doi: 10.3390/pathogens10020250 (PMC7926304; doi:10.3390/pathogens10020250)
Supplement: Supplementary file 1 [file pathogens-10-00250-s001.zip › pathogens-1079095-supplementary/pathogens-1079095-supplementary.docx]

## Supplementary Data

**Table S1.** Perception & understanding of veterinarians (*n* = 15).

| Variables | Strongly agree | | Agree | | Undecided | | Disagree | | Strongly disagree | |
| --- | --- | --- | --- | --- | --- | --- | --- | --- | --- | --- |
|  | *n* | % | *n* | % | *n* | % | *n* | % | *n* | % |
| My contribution or job having responsibility to control zoonotic diseases | 9 | 60.0 | 2 | 13.3 | 3 | 20.0 | 1 | 6.7 | - | - |
| I have enough confidence to diagnose, identify and differentiate parasitic diseases | - | - | 1 | 6.7 | 5 | 33.3 | 3 | 20.0 | 6 | 40.0 |
| Captive wildlife parasite can be transmitted to human/animal from environment and vice-versa | 4 | 26.7 | 8 | 53.3 | 3 | 20.0 | - | - | - | - |
| I have good understanding in protecting myself from zoonoses | - | - | 3 | 20.0 | 6 | 40.0 | 6 | 40.0 | - | - |
| Do you consider that the one health can be effective to control parasitic diseases | 5 | 33.3 | 8 | 53.3 | 2 | 13.3 | - | - | - | - |
| There are enough resources to learn one health | - | - | 3 | 20.0 | 1 | 6.7 | 5 | 33.3 | 6 | 40.0 |
| I have sound understanding of antiparasiticidal-resistant | - | - | 1 | 6.7 | 2 | 13.3 | 5 | 33.3 | 7 | 46.7 |
| One health concept should be promoted by national and local government in Bangladesh | 9 | 60.0 | 2 | 13.3 | 3 | 20.0 | 1 | 6.7 | - | - |
